# Curcumin suppresses cell proliferation and reduces cholesterol absorption in Caco-2 cells by activating the TRPA1 channel

**DOI:** 10.1186/s12944-022-01750-7

**Published:** 2023-01-14

**Authors:** Si Qin, Qian Su, Xiang Li, Muqing Shao, Yindi Zhang, Fadong Yu, Yinxing Ni, Jian Zhong

**Affiliations:** 1 Department of Endocrinology, The Third Affiliated Hospital of Chongqing Medical University, No.1, Shuanghu Branch Road, Chongqing, 401120 China; 2 College of Chemistry and Molecular Engineering, Peking University, 100871 Beijing, China

**Keywords:** Cholesterol absorption, Curcumin, Caco-2 cells, NPC1L1, Proliferation, TRPA1

## Abstract

**Background:**

Curcumin (Cur) is a bioactive dietary polyphenol of turmeric with various biological activities against several cancers. Colorectal cancer (CRC) is one of the leading causes of cancer-related deaths. Intestinal cholesterol homeostasis is associated with CRC. Chemotherapy for CRC is related to varied adverse effects. Therefore, natural products with anti-cancer properties represent a potential strategy for primary prevention of CRC.

**Methods:**

The present study used Cur as a therapeutic approach against CRC using the Caco-2 cell line. The cells were treated with different concentrations of Cur for different duration of time and then the proliferation ability of cells was assessed using Cell Counting Kit-8 and 5-Ethynyl-2′-deoxyuridine assays. Oil red O staining and cholesterol assay kit were used to evaluate cellular lipid content and cholesterol outward transportation. Finally, the protein expressions of cholesterol transport-related protein and signal transduction molecules were assessed using Western blot assay.

**Results:**

Cur inhibited cell proliferation in Caco-2 cells in a dose- and time-dependent manner by activating the transient receptor potential cation channel subfamily A member 1 (TRPA1) channel. Activation of the TRPA1 channel led to increased intracellular calcium, peroxisome proliferator-activated receptor gamma (PPARγ) upregulation, and the subsequent downregulation of the specificity protein-1 (SP-1)/sterol regulatory element-binding protein-2 (SREBP-2)/Niemann-Pick C1-like 1 (NPC1L1) signaling pathway-related proteins, and finally reduced cholesterol absorption in Caco-2 cells.

**Conclusions:**

Cur inhibits cell proliferation and reduces cholesterol absorption in Caco-2 cells through the Ca^2+^/PPARγ/SP-1/SREBP-2/NPC1L1 signaling by activating the TRPA1 channel, suggesting that Cur can be used as a dietary supplement for the primary prevention of CRC.

**Graphical Abstract:**

In Caco-2 cells, Cur first stimulates calcium influx by activating the TRPA1 channel, further upregulates PPARγ and downregulates SP-1/SREBP-2/NPC1L1 signaling pathway, and finally inhibits the absorption of cholesterol. TRPA1, transient receptor potential cation channel subfamily A member 1; NPC1L1, Niemann-Pick C1-like 1; PPARγ, peroxisome proliferator-activated receptor gamma; SP-1, specificity protein-1; SREBP-2, sterol regulatory element-binding protein-2; Cur, curcumin.

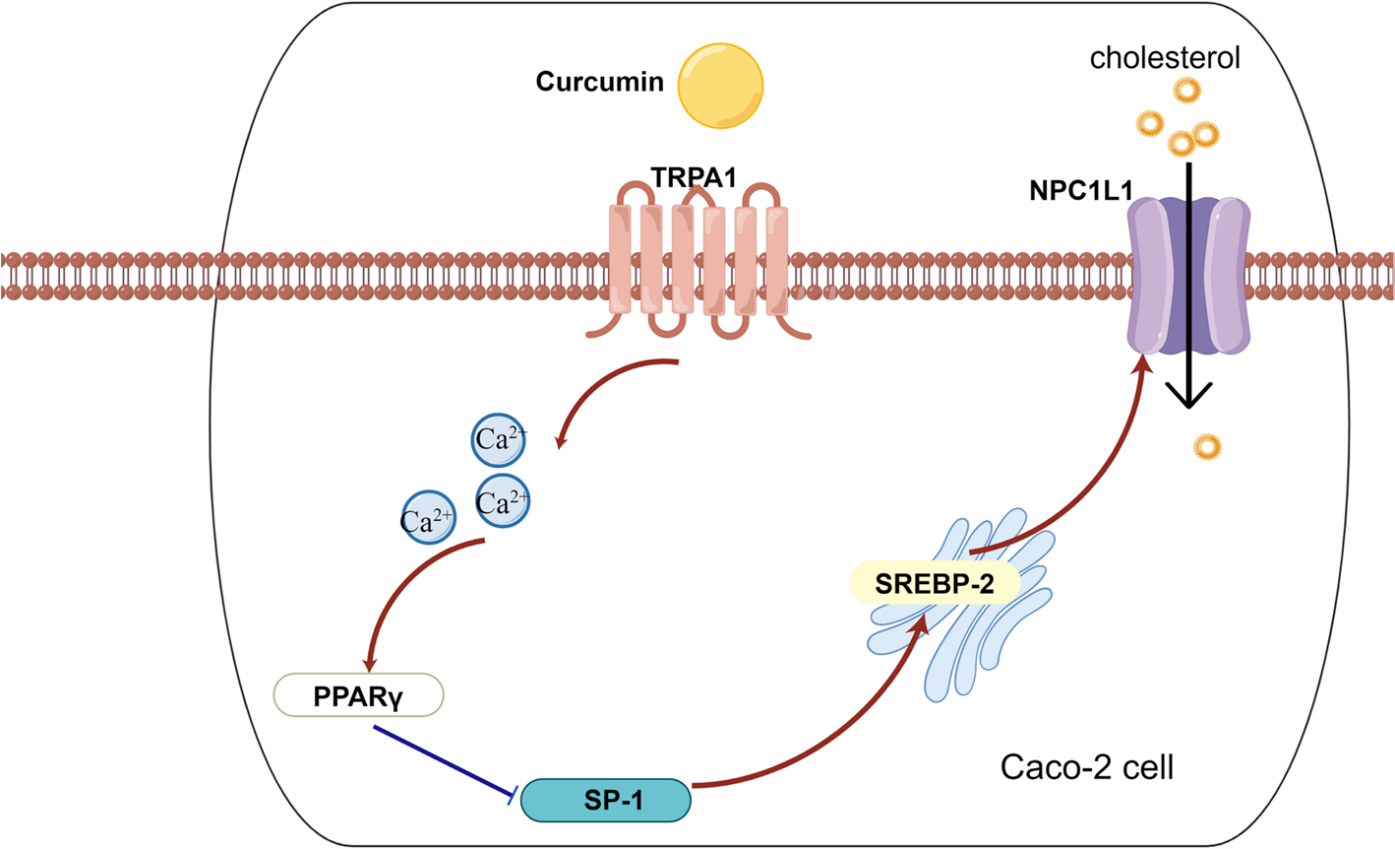

**Supplementary Information:**

The online version contains supplementary material available at 10.1186/s12944-022-01750-7.

## Introduction

Colorectal cancer (CRC), the third most common form of cancer, ranks as the second leading cause of cancer-related deaths worldwide [1]. Genetic factors, living habits, diet, obesity, and dysregulation of gut microbes are traditional risk factors for CRC [2–4]. Recently, abnormal lipid metabolism in the gut has been determined to be a hallmark characteristic associated with the pathogenesis and progression of CRC [1]. On one hand, cholesteryl ester, a storage form lipid in enterocytes, could correlate to the pathogenesis, progression and disparities in CRC [5]. On the other hand, intestinal cholesterol accumulation also aggravates CRC progression and metastasis [6]. Therefore, inhibition of the gut cholesterol absorption may reduce the risk of CRC.

Absorption of intestinal cholesterol mainly includes three key steps. First, dietary cholesterol is micellized by bile acids in the bowel lumen; then, it is absorbed by the intestinal epithelial cells (IECs) and assembled into lipoproteins; finally, it is transported to the lymph and the blood circulation [7]. Importantly, intestinal cholesterol absorption in the IECs is principally mediated by Niemann-Pick C1-like 1 (NPC1L1) [8]. Ezetimibe, an inhibitor of NPC1L1-mediated cholesterol absorption, could effectively improve hypercholesterolemia [9, 10]. However, this drug could not be used as primary prevention for hypercholesterolemia and CRC. Current options for treating CRC are surgery, chemotherapy, radiotherapy, and tumor immunotherapy [11], but each of these is associated with various adverse effects and high costs. Therefore, it is crucial to develop natural agents for the primary prevention of CRC as a complementary option as these are affordable and associated with fewer side effects.

**Fig. 1:**
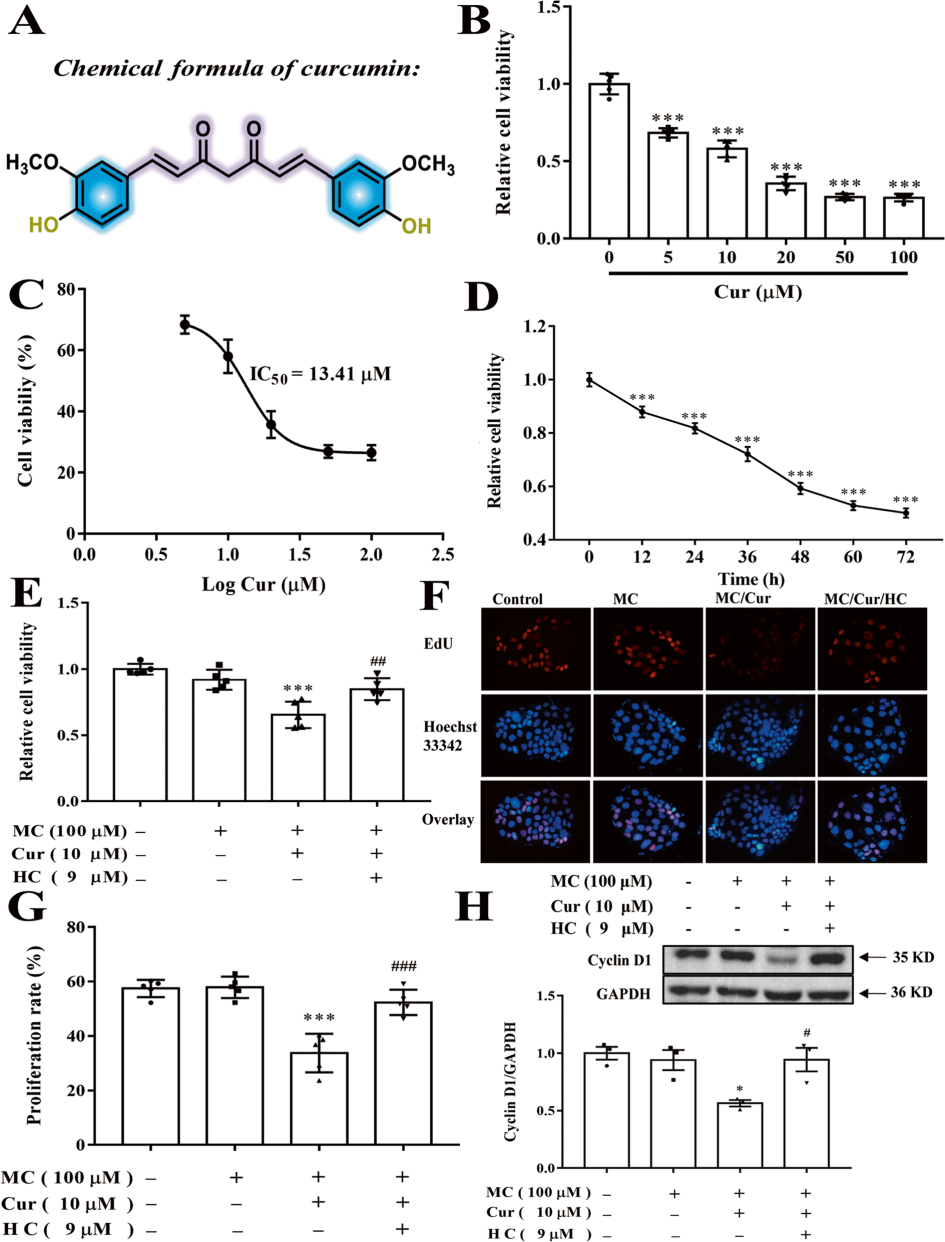
Effect of Cur on Caco-2 cell proliferation by activating the TRPA1 channel. **A** Chemical structure formula of Cur. **B** Caco-2 cells incubated with different concentrations of Cur (0, 5, 10, 20, 50, and 100 µM) for 48 h. **C** Cell viability of Caco-2 cells after treatment with different concentrations of Cur and the corresponding IC_50_ values. **D** Caco-2 cells were incubated with Cur (10 µM) for different durations (0, 12, 24, 36, 48, 60, and 72 h). **E**, **F**, **G** and **H** Caco-2 cells were incubated with the indicated concentrations of MC (100 µM), Cur (10 µM), and HC (9 µM) for 48 h. Cell viability assessed by CCK-8 assay (**B**, **C**, **D** and **E**). Representative EdU image by fluorescence microscopy (magnification, 400×) (**F**) and quantification of proliferation rate (**G**). Cyclin D1 protein expression was determined by Western blot analysis (**H**). Data presented as mean ± SEM. (**B**, **C**, **D**, **E**, **F**, **G**, *n* = 5), (**H**, *n* = 3). ^*^P < 0.05 vs MC (**H**), ^***^P < 0.001 vs control (**B**, **D**) or MC (**E**, **G**), ^#^*P* < 0.05 vs MC + Cur (**H**), ^##^*P* < 0.01 vs MC + Cur (**E**), ^###^*P* < 0.001 vs MC + Cur (**G**). Cur, curcumin; MC, micellar cholesterol; HC, HC-030031; EdU, 5-Ethynyl-2′-deoxyuridine; IC_50_, inhibitory concentration 50; CCK-8, Cell Counting Kit-8; TRPA1, transient receptor potential cation channel subfamily A member 1; Cyclin D1, cyclin dependent kinase 1; SEM, standard error of the mean

Curcumin (Cur) [(E,E)-1,7-Bis(4-hydroxy-3-methoxyphenyl)-1,6-heptadiene-3,5-dione] (Fig. 1A), a bioactive dietary polyphenol of turmeric [12], has a broad range of biological functions [13, 14]. Several studies have demonstrated the protective effects of Cur against varied cancers, including lung cancer, breast cancer, gastric cancer, CRC and so on [15, 16]. In addition, an epidemiological analysis showed an association between dietary consumption of Cur and decreased incidence of CRC in the Indian population [17]. Till date, several potential mechanisms of Cur-related anti-cancer effects in CRC have been identified, such as mechanisms involving DNA damage, cell cycle arrest, apoptosis, CRC angiogenesis, epidermal growth factor receptor signaling pathway, and tumor immunomodulation [18]. However, the accurate mechanism of Cur and its key regulatory protein involved in inhibiting CRC cell proliferation in a high-fat diet setting remains to be confirmed. Furthermore, Zou J. *et al* found that Cur could reduce intestinal cholesterol absorption by 51% in mice [19]. On one hand, the study has shown that Cur could regulate the peroxisome proliferator-activated receptor gamma (PPARγ) /specificity protein-1 (SP-1) /sterol regulatory element-binding protein-2 (SREBP-2) signaling pathway in activated hepatic stellate cells (HSCs) [20]. On the other hand, Cur was shown to control the transcription of NPC1L1 via SREBP-2 in hamsters [21], and finally reduce intestinal cholesterol absorption. Furthermore, Cur was shown to activate the transient receptor potential cation channel subfamily A member 1 (TRPA1) channel in HEK293 cells to increase the intracellular calcium concentrations [22, 23], and then the increased calcium up-regulated PPARγ expression [24].

Caco-2 cell model, a clone of colorectal adenocarcinoma cells from human, is a classical *in vitro* model to identify formulations that affect intestinal cholesterol transport [25, 26]. Enterocytic cells were shown to differentiate from Caco-2 cells upon attaining confluence [27]. Then, after detecting *NPC1L1* expression levels in various CRC cell lines, *NPC1L1* is highly expressed in both Caco-2 and SW620 cell line [28]. However, *TRPA1* is only expressed in Caco-2 cell line, but not in SW620 cell line [29]. Thus, Caco-2 cells were chosen for the present study.

The purpose of this study was to examine the influence of Cur on cholesterol absorption and cell proliferation in Caco-2 cells and to investigate the underlying molecular mechanism. The present study suggested that Cur could inhibit cell proliferation and reduce cholesterol absorption via regulation of the Ca^2+^/PPARγ/SP-1/SREBP-2/NPC1L1 signaling pathway by TRPA1 activation in Caco-2 cells, and that Cur has potential for use in the primary prevention of CRC.

## Material and methods

### Cell culture and treatment

Stable Caco-2 cell lines, which were derived from colorectal adenocarcinoma, were purchased from Procell (Wuhan, China) and certificated by American Type Culture Collection. The cells were cultured in Dulbecco's Modified Eagle Medium (DMEM) containing 4.5 g/L Glucose (Gibco, Thermo Fisher Scientific, Shanghai, China) supplemented with 10% fetal bovine serum (Biological Industries, Beijing, China), 1% penicillin-streptomycin (Beyotime, Shanghai, China), at 37 °C in a humidified environment containing 5% CO_2_. In six-well Falcon plates, the cells were plated at a density of 2.5 × 10^4^ cells/cm^2^, and fresh culture media was added every alternate day [30]. Cur was supplied by Sigma (St. Louis, MO, USA). Cur was diluted to 20 mM in dimethyl sulfoxide and stored at − 20 °C for experimental treatments.

### Preparation of cholesterol micelles

Stock solutions of taurocholate (Solarbio, Beijing, China) were dissolved in methanol; stock solutions of egg yolk phosphatidylcholine (Solarbio, Beijing, China) and cholesterol (Solarbio, Beijing, China) were dissolved in chloroform. Each stock solution was mixed in the ratio of 50:1:10 (W: W: W) in a vortex and then transferred to a glass tube that could be sealed. A mild stream of N_2_ evaporated the solvents before lyophilization for 8–16 h [31]. The lipid film was stored at − 20 ℃. About two hours before the experiment began, the lipid film was moistened with serum-free DMEM and incubated at 37 ℃ [32]. Before adding the solutions to the cells, they were filtered through a 0.45 µM cellulose acetate filter.

### Cell proliferation assay

Cell Counting Kit-8 (CCK-8) and 5-Ethynyl-2′-deoxyuridine (EdU) assay were used to detect the proliferation ability of Caco-2 cells. For CCK-8 assay, Caco-2 cells were cultured at a density of 5,000 cells/well in 96-well plates at 37 °C, in a humid atmosphere containing 5% CO_2_. After corresponding treatment, when the cells had achieved 70% confluence, each well was filled with 100 µL of fresh medium and 10 µL of CCK-8 (Beyotime, Shanghai, China) solution and then incubated for 2 h [33]. The absorbance of each well at 450 nm was measured using a microplate reader (Bio-Rad, Hercules, CA, USA).

For EdU assay, logarithmic growth stage cells were seeded in 12-well plate, and incubated with 10 µM EdU from a Click-tm EdU Cell Proliferation Kit [34] (Beyotime, Shanghai, China) for 2 h at 37 °C after corresponding treatment. The cells were permeabilized with 0.3% Triton X-100 for 20 min after incubating with 4% paraformaldehyde for 20 min. After washing thrice with phosphate-buffered saline (PBS) for 5 min each, the cells were dyed with click additive solution for 2 h. Caco-2 nuclei stained with Hoechst-33342 were used for the cell counts, and the relative EdU-positive ratio was examined under a fluorescence microscope (ECLIPSE Ti; Nikon, Tokyo, Japan) and quantified by Image J software (National Institutes of Health, Bethesda, MD).

### Oil red O staining and quantification of lipid content

Modified Oil Red O Staining Kit (Beyotime, Shanghai, China) was used to access neutral lipids in Caco-2 cells in accordance with the manufacturer's instructions. The cells were washed three times with PBS and then fixed with 4% Paraformaldehyde at 4 ℃ for 15–30 min. After the fixative was removed, the samples were rinsed with PBS and stained with oil red O. After 30–60 min, the oil red O was removed, and the samples were rinsed with scrubbing solution and double-distilled water. The image was accessed using a full-automatic scanning imaging microscope (original magnification, 200×/400×) with a Viewpoint Light photographic attachment. To further quantify the lipid content, the bound dye was dissolved in isopropyl alcohol and monitored spectrophotometrically by reading the absorbance at 510 nm [35]. The cells were then scraped and lysed, and the protein concentration was determined using a bicinchoninic acid (BCA) assay by using a Beyotime kit (Shanghai, China). Absorbance was calculated for the protein concentration per plate.

### Cellular cholesterol transportation

The cholesterol levels in cells and culture medium were assayed using a liquid and tissue total cholesterol assay kit (Applygen Technologies Inc., Beijing, China). Experimental protocols were followed as directed by the manufacturer. Cellular protein content was measured based on a BCA assay using a Beyotime kit (Shanghai, China). Outward cholesterol transport rate of cells = cholesterol concentration of culture supernatant/ (cholesterol concentration of culture supernatant + total cholesterol in cells) × 100%.

## Measurement of Ca^2+^ signals/Ca^2+^ influx

According to the manufacturer's instructions, intracellular Ca^2+^ levels were measured with a Fluo-4 AM calcium assay kit (Beyotime, Shanghai, China). The procedure was as follows: cells were seeded into black 96-well plates at a density of 5 × 10^4^ cells per mL. Experimental groups were pretreated with 9 µM TRPA1 antagonist- HC-030031 (Selleck, USA) for 30 minutes. Cells were then washed thrice with Ca^2+^-free bath solution and stained with 5 µM Fluo-4 AM for 60 minutes at 37 °C in the dark, which allowed the cleaving of AM esters by cellular esterases after crossing the plasma membrane. This rendered the dye cell impermeant and was thus retained inside the cells. Cells were then washed three times with Hank’s Balanced Salt Solution containing CaCl_2_, and the basic fluorescence level was measured at excitation/emission wavelengths of 488/516 nm for 3 min using a fluorescence microplate reader (Thermo Scientific, Shanghai, China) [36]. Next, all groups were exposed to Cur for 12 min, and the Ca^2+^ influx was continued to be measured by estimating the actual ratio of fluorescence intensity divided by the mean baseline fluorescence intensity. Statistical analysis was performed using independent experiments with three batches of cell culture.

### Western blot analysis

The proteins expression levels of TRPA1, NPC1L1, PPARγ, SP-1, and SREBP-2 were examined by Western blot assay. Protein expression of cyclin dependent kinase 1 (Cyclin D1), one of the targeting proteins specific to cancer involved in cell cycle progression and proliferation [37], was also determined. Briefly, proteins were extracted from the cells in each group by RIPA lysis buffer (Beyotime, Shanghai, China), following which the concentration was determined using a BCA protein assay kit (Beyotime, Shanghai, China). The protein samples were then loaded onto an 8% or 10% sodium dodecyl sulfate-polyacrylamide gel electrophoresis and subsequently electro-transferred to NC transfer membranes. A 5% non-fat milk solution in Tris-buffered saline with Tween-20 (TBST) was used to block the membrane for one hour. After washing three times, the membrane was incubated with primary antibodies at 4 °C overnight, followed by appropriate secondary antibodies for one hour at room temperature. After the membranes were washed in TBST, proteins bound complexes were detected using the Odyssey Infrared Imaging System (Li-Cor Biosciences, Lincoln, NE), and the band intensities were quantified by densitometry using Image J.

### The Cancer Genome Atlas (TCGA) data of NPC1L1 expression in human CRC tissues

Normalized gene expression data for TCGA Pan-Cancer (PANCAN, Samples = 10535, Genes = 60499) were first obtained from the University of California Santa Cruz (https://xenabrowser.net/). Next, *NPC1L1* (ENSG00000015520) expression data in colon adenocarcinoma (COAD) and colon adenocarcinoma/rectum adenocarcinoma/esophageal carcinoma (COADREAD) were extracted and transformed by Log_2_ (transcripts per million + 0.001). Finally, statistical significance analysis of *NPC1L1* expression in the normal and cancer tissues (*P* value for COAD: 7.1x10^− 8^, *P* value for COADREDA: 1.8x10^–8^) was performed using unpaired Wilcoxon rank sum and signed rank tests by R package (version 3.6.4).

### Statistical analysis

Within each experiment, each condition or treatment was performed in a minimum of triplicates. Data were expressed as the average ± standard error of the mean (SEM). GraphPad Prism version 7 (GraphPad Software, San Diego, CA) was used to analyze differences between samples, either by the two-sample Student’s *t* test or by analysis of variance with Newman-Keuls multiple test for differences between selected pairs of samples. *P* values of 0.05 were considered statistically significant.

## Results

### Cur inhibited the proliferation of Caco-2 cells via the TRPA1 channel

To explore the effects of Cur on Caco-2 cell proliferation, Caco-2 cells were treated with various concentrations of Cur (5, 10, 20, 50, and 100 µM) for 48 h. Cellular morphology and growth were observed daily under an inverted microscope, and cell proliferation was identified by CCK-8 assay and EdU assay. Cur notably decreased the proliferation in a concentration-dependent manner (Supplemental Fig. 1A), and only ~ 30% cells survived after 100 µM Cur treatment (Fig. 1B). The concentration of Cur exerting a half-maximal increase in intracellular calcium was 3.3 µM in HEK293 cells [23], and the inhibitory concentration 50 (IC_50_) of Cur was approximately 13.41 µM (Fig. 1C). Therefore, a low-toxic concentration of 10 µM was used for further investigation. In addition, Cur at a concentration of 10 µM can inhibit cell proliferation in a time-dependent manner (Fig. 1D). Considering that abnormal intestinal lipid metabolism is closely related to the occurrence and development of CRC [1]. For further study, Caco-2 cells were pre-incubated for 24 h with micellar cholesterol (MC) to make a high-cholesterol environment. Similarly, co-treatment with 10 µM Cur also markedly inhibited the proliferation of Caco-2 cells while MC incubation without Cur almost did not affect cell proliferation (Supplemental Fig. 1B). Importantly, it can be further noted that the inhibitory effects of Cur on Caco-2 cell proliferation were related to the activation of the TRPA1 channel. Results of CCK-8 assay (Fig. 1E) and EdU assay (Fig. 1F and 1G) showed that HC-030031 (HC), a potent and selective TRPA1 inhibitor, efficiently blocked the anti-proliferative effects of Cur on Caco-2 cells at a concentration of 9 µM (the same concentration used in the experiment about the inflammatory role of TRPA1 in lung epithelial cells [38]). Moreover, Cur treatment significantly downregulated protein expression of the cell cycle regulator, Cyclin D1, and this inhibitory effect was reversed by HC (Fig. 1H). Collectively, these data suggested that Cur inhibited the proliferation of Caco-2 cells, possibly by facilitating TRPA1 channel activation.

### Cur attenuated intracellular lipid accumulation of Caco-2 cells via the TRPA1 channel

**Fig. 2:**
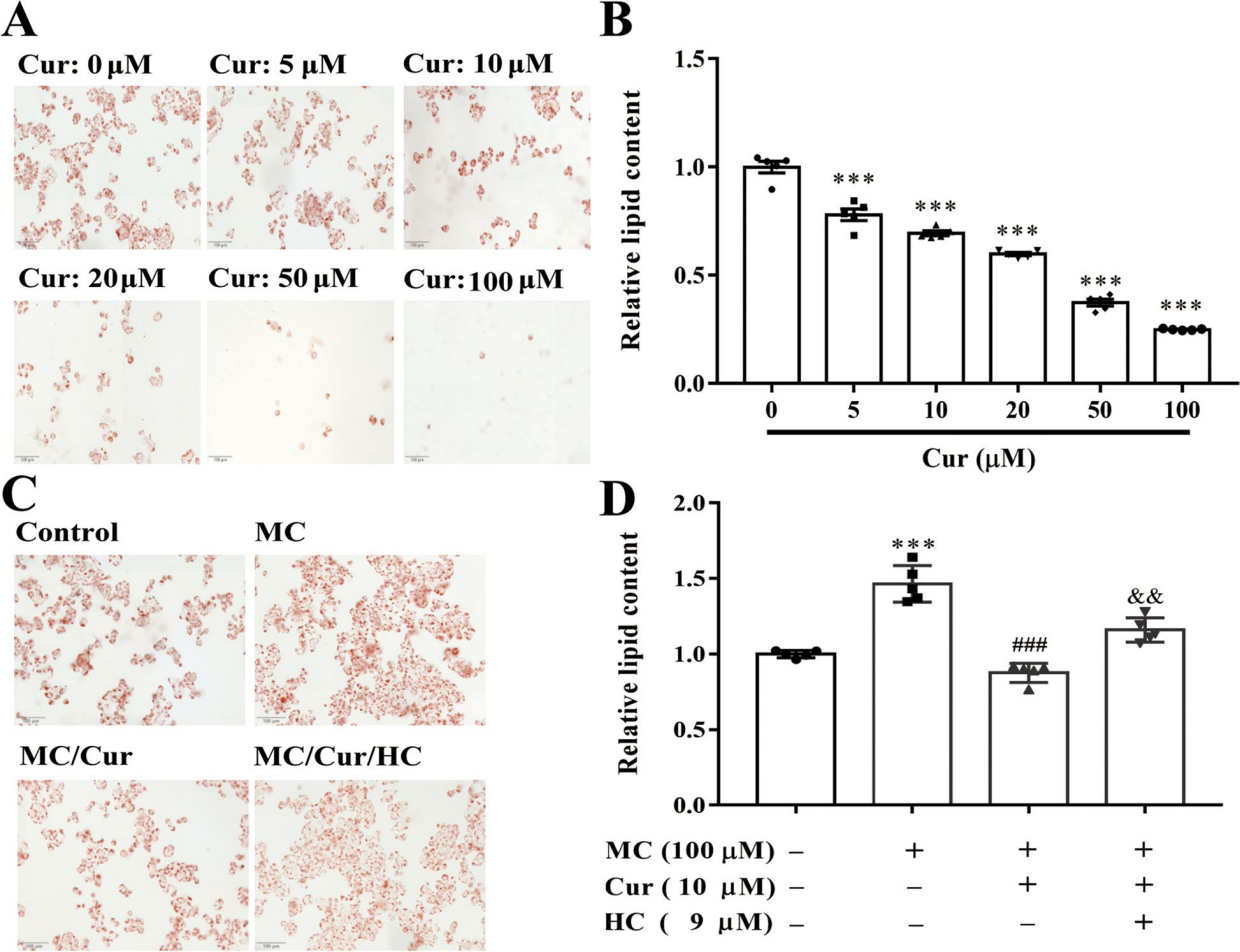
Effect of Cur on intracellular lipid accumulation in Caco-2 cells by activating the TRPA1 channel. **A** and **B** Caco-2 cells were incubated with different concentrations (0, 5, 10, 20, 50, and 100 µM) of Cur for 48 h. **C** and **D** Cells of the Caco-2 line were incubated with the indicated concentrations of MC (100 µM), Cur (10 µM), and HC (9 µM) for 48 h. **A** and **C** Oil red O staining images of Caco-2 cells (magnification 200 ×). **B** and **D** Quantification of lipid content by a microplate reader. Data presented as mean ± SEM. (*n* = 5). ^***^P < 0.001 vs control, ^###^*P* < 0.001 vs MC, ^&&^*P* < 0.01 vs MC + Cur. Cur, curcumin; MC, micellar cholesterol; HC, HC-030031; TRPA1, transient receptor potential cation channel subfamily A member 1; SEM, standard error of the mean

Next, for further study of cholesterol absorption, cell-climbing films were stained with oil red O staining to evaluate intracellular lipid content. As shown in Fig. 2A, Cur attenuated the lipid uptake in a concentration-dependent manner in Caco-2 cells. Notably, after pre-incubating with MC for 24 h, a large number of red lipid droplets accumulated in the cells. However, 10 µM of Cur effectively arrested lipid droplets compared with the group that received MC alone, and this inhibitory effect was reversed by HC (Fig. 2C). Furthermore, these findings were consistent with the results of the lipid content analysis with a microplate reader (Fig. 2B and D), which indicated that Cur might reduce lipid deposition in Caco-2 cells by activating the TRPA1 channel.

### Cur, via activating the TRPA1 channel, promoted intracellular cholesterol transport of Caco-2 cells

**Fig. 3:**
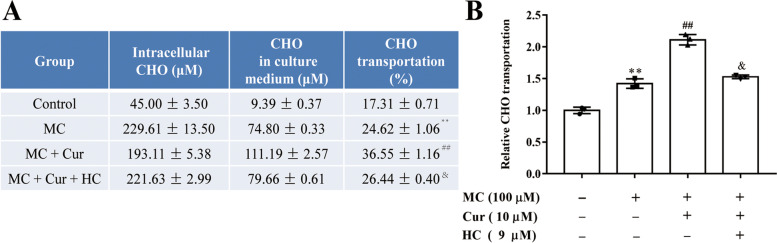
Effects of Cur on outward cholesterol transport rate in Caco-2 cells by activating the TRPA1 channel. **A** and **B** Caco-2 cells were incubated with the indicated concentrations of MC (100 µM), Cur (10 µM), and HC (9 µM) for 48 h. **A** Table of cholesterol (CHO) content and transport rate in Caco-2 cells and supernatant of the culture medium in each group. **B** Relative CHO transport rate in each group. Data presented as mean ± SEM. (*n* = 3). ^**^P < 0.01 vs control, ^##^*P* < 0.01 vs MC, ^&^*P* < 0.05 vs MC + Cur. CHO, cholesterol; MC, micellar cholesterol; Cur, curcumin; HC, HC-030031. TRPA1, transient receptor potential cation channel subfamily A member 1; SEM, standard error of the mean

Then, to explore cholesterol transport more specifically in Caco-2 cells, the cholesterol concentrations in Caco-2 cells and culture medium were quantified from different groups. The results of the analysis are illustrated in Fig. 3A. Compared with the control group, micelle-treated cells showed an obvious increase in cholesterol accumulation both in cells and culture medium. Compared to the micelles group, the outward cholesterol transport rate was markedly elevated in Caco-2 cells co-incubated with MC and 10 µM Cur, which was reversed in the cells treated with micelles, Cur and HC at the same time (Fig. 3B), suggesting that Cur could decrease cholesterol absorption in Caco-2 cells by activating the TRPA1 channel.

### Cur, via activating the TRPA1 channel, mediated calcium influx of Caco-2 cells

**Fig. 4:**
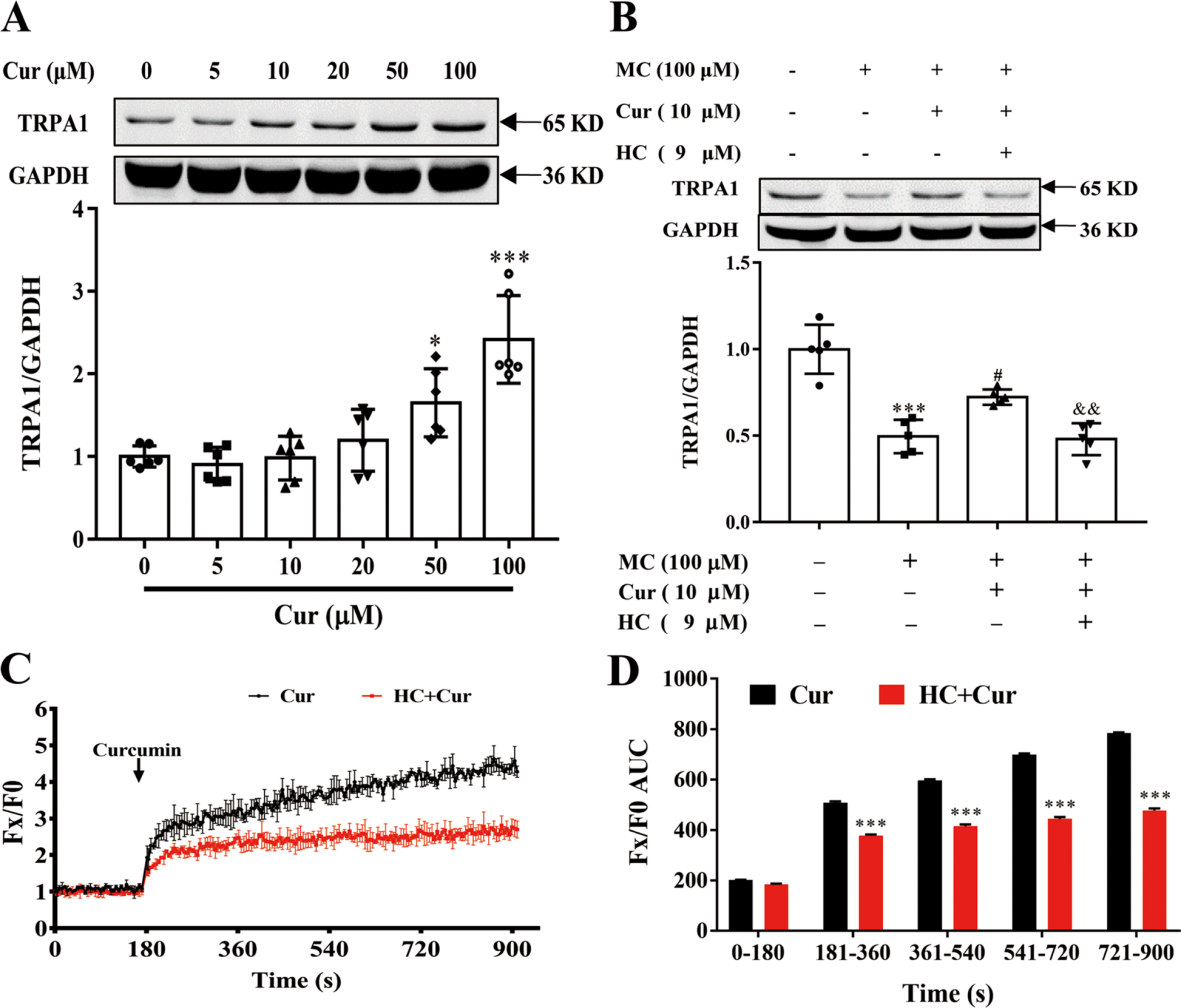
Effect of Cur on TRPA1 protein expression and calcium influx in Caco-2 cells by activating the TRPA1 channel. **A** Caco-2 cells were incubated with different concentrations of Cur (0, 5, 10, 20, 50, and 100 µM) for 48 h, and TRPA1 protein expression was then determined by Western blotting (*n* = 6). **B** Caco-2 cells were then incubated with the indicated concentrations of MC (100 µM), Cur (10 µM), and HC (9 µM) for 48 h, and Western blotting was used to detect TRPA1 protein expression (*n* = 5). **C** Intracellular Ca^2+^ levels in Caco-2 cells exposed to Cur with or without HC exposure were detected by a Fluo-4 AM calcium assay kit (*n* = 3). **D** AUC of intracellular Ca^2+^ levels at different time periods. Data presented as mean ± SEM. ^*^P < 0.05 vs control, ^***^P < 0.001 vs control or Cur (**D**), ^#^*P* < 0.05 vs MC, ^&&^*P* < 0.01 vs MC + Cur. Cur, curcumin; TRPA1, transient receptor potential cation channel subfamily A member 1; GAPDH, glyceraldehyde-3-phosphate dehydrogenase; MC, micellar cholesterol; HC, HC-030031; AUC, area under curve; SEM, standard error of the mean

Studies once have reported that Cur could activate the TRPA1 channel in HEK293 cells and increase intracellular calcium concentration [22, 23]. To further determine whether Cur could promote calcium influx to reduce cholesterol absorption via activation of the TRPA1 channel in Caco-2 cells, the TRPA1 protein levels of Caco-2 cells in each group were detected by Western blot analysis. The results demonstrated that Cur significantly increased TRPA1 protein expression in a concentration-dependent manner starting from 50 µM, and this effect was most obvious at 100 µM of Cur (Fig. 4A). In addition, Cur also rescued the MC-induced decrease in TRPA1 expression, which was blocked by the TRPA1 inhibitor HC (Fig. 4B). At Cur concentration of 50 µM, the growth inhibition rate of Caco-2 cells was at least as high as 70%, so a relatively low-toxic concentration of 20 µM was used for the investigation. Next, to verify whether Cur could perturb intracellular Ca^2+^ homeostasis via TRPA1 channels, Fluo-4 AM (a cytosolic Ca^2+^ indicator) was prelabeled Caco-2 cells with 20 µM Cur at 180 s, and Fluo-4 AM fluorescence was monitored within 900 s. As shown in Fig. 4C, Cur stimulation significantly evoked Ca^2+^ signals in the cytosol of Caco-2 cells. However, incubation with HC for 0.5 h could partially inhibit this calcium influx. Similarly, the area under curve (AUC) of group preincubation with HC was significantly lower than that of Cur group at different time periods (Fig. 4D).

### Regulation of Cur on cholesterol transport-related proteins expressions: role of PPARγ activation

**Fig. 5:**
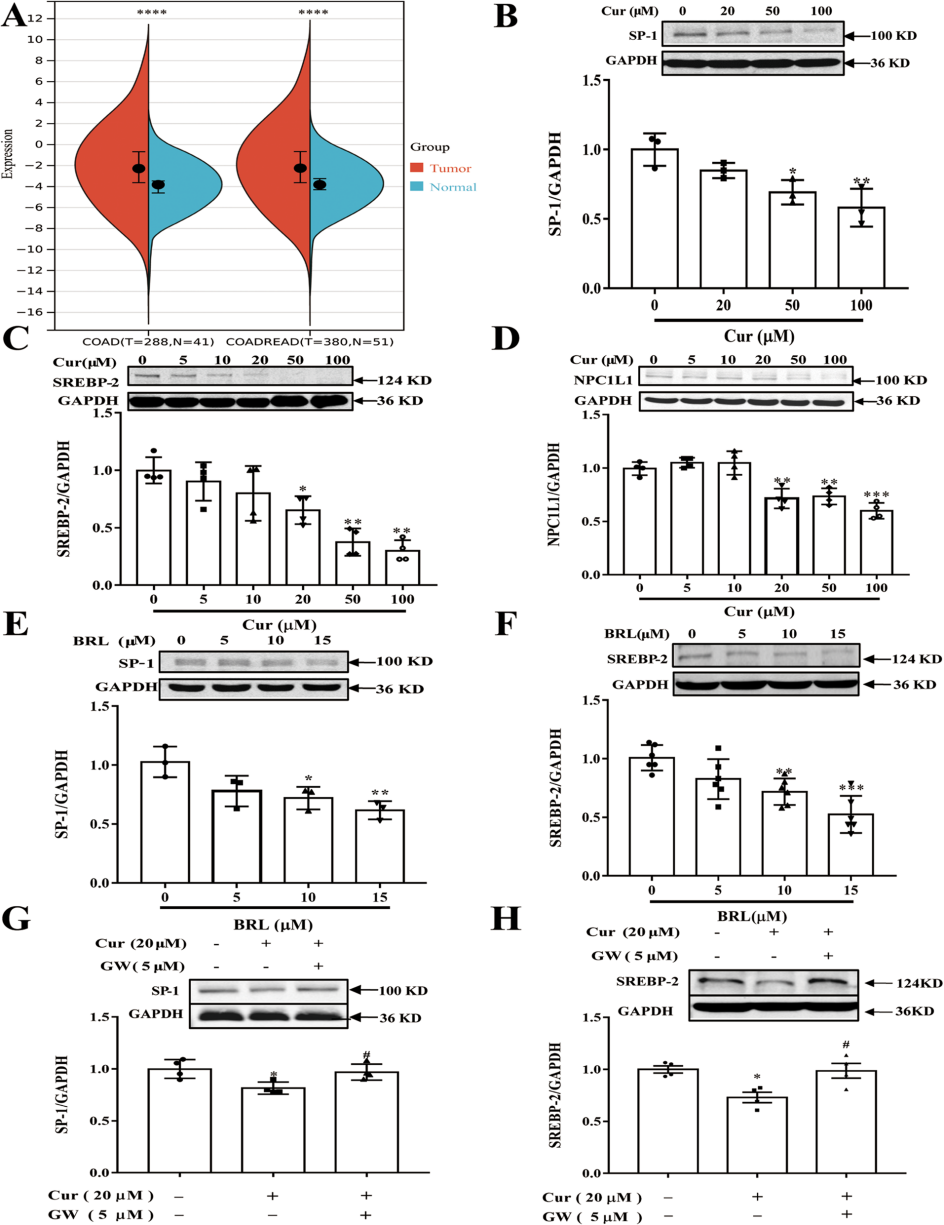
Effect of Cur on the expression levels of SP-1, SREBP-2, and NPC1L1 proteins and the activation of PPARγ resulted in the suppression of the SP-1 and SREBP-2 proteins in Caco-2 cells. **A** Based on TCGA database, NPC1L1 expression was higher in CRC tissues. **B**, **C**, and **D** Caco-2 cells were incubated with different concentrations of Cur for 48 h, and the expression levels of the SP-1 (**B**), SREBP-2 (**C**), and NPC1L1 (**D**) proteins were determined by Western blot analysis. (*n* = 3, 4, and 7). **E** and **F** Caco-2 cells were incubated with various concentrations (0, 5, 10, and 15 µM) of PPARγ agonist BRL for 48 h, and the expression of SP-1 (**E**) and SREBP-2 (**F**) proteins were detected by Western blot analysis. (*n* = 3 and 6). **G** and **H** Cells of the Caco-2 line were incubated with the indicated concentrations of Cur (20 µM) and PPARγ antagonist GW (5 µM) for 48 h, and the expression levels of SP-1 and SREBP-2 proteins were determined by Western blot analysis. (*n* = 4). Data presented as mean ± SEM. ^*^P < 0.05 vs control, ^**^P < 0.01 vs control, ^***^P < 0.001 vs control, ^#^*P* < 0.05 vs Cur, ^##^*P* < 0.01 vs Cur. Cur, curcumin; SP-1, specificity protein-1; GAPDH, Glyceraldehyde-3-phosphate dehydrogenase; SREBP-2, sterol regulatory element-binding protein-2; NPC1L1, Niemann-Pick C1-like 1; BRL, rosiglitazone, BRL 49653; GW, GW 9662; PPARγ, peroxisome proliferator-activated receptor gamma; TCGA, the Cancer Genome Atlas; CRC, colorectal cancer; SEM, standard error of the mean

Cholesterol absorption in the IECs is mainly mediated by NPC1L1 [8], and TCGA, a database of molecular signatures collected from human cancer tissues, showed that NPC1L1 was more highly expressed in human CRC rather than in normal tissues (*p* ＜ 0.0001) (Fig. 5A). Furthermore, NPC1L1 expression is modulated by SP-1/SREBP-2 [30], the protein levels of SP-1 and SREBP-2 were significantly decreased along with increasing concentrations of Cur (Fig. 5B and C). Besides, NPC1L1 expression was downregulated in Caco-2 cells treated with Cur in a concentration-dependent manner (Fig. 5D). Furthermore, studies have shown that Cur could regulate the PPARγ/SP-1/SREBP-2 signaling pathway in HSCs [20]. To test whether PPARγ is also involved in regulating the levels of SP-1 and SREBP-2 in Caco-2 cells, cells were incubated with various concentrations of the PPARγ agonist for 48 h, and then the whole-cell extracts were obtained. As shown in Fig. 5E and F by Western blot analyses, compared with the untreated control (first well), rosiglitazone (BRL 49653; BRL, Cayman Chemical, Ann Arbor, Michigan) significantly decreased protein abundances of SP-1 and SREBP-2. In Caco-2 cells, the blockade of PPARγ activation by antagonist GW 9662 (Cayman Chemical, Ann Arbor, Michigan) attenuated the inhibitory effects of Cur on the SP-1 and SREBP-2 protein abundances (Fig. 5G and H), suggesting the requirement of PPARγ activation in this process.

### Cur activated PPARγ and regulated cholesterol transport-related protein expression via the TRPA1 channel in a high-cholesterol models

**Fig. 6:**
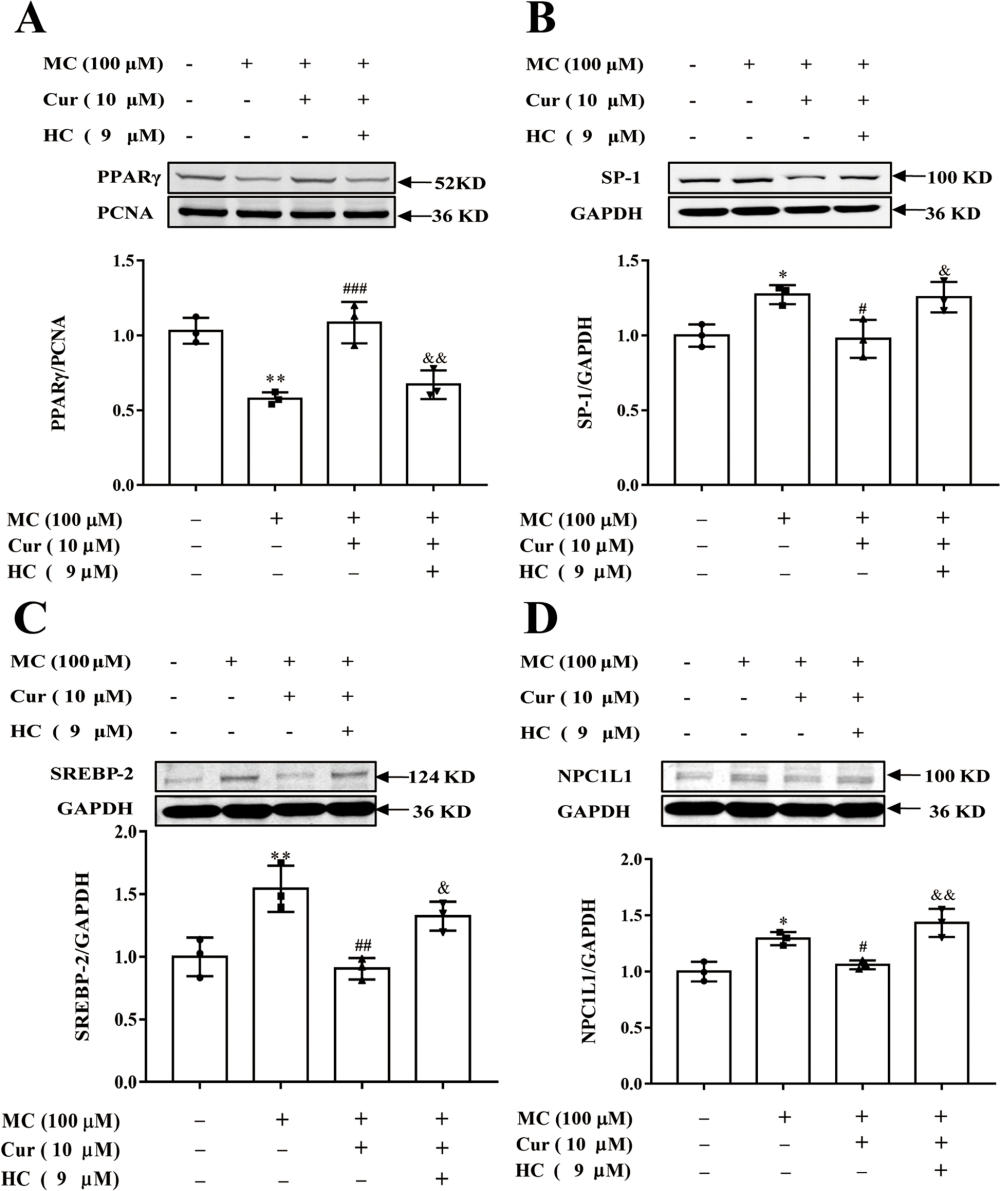
Effect of Cur on the PPARγ/SP-1/SREBP-2/NPC1L1 signaling pathway in Caco-2 cells by activating the TRPA1 channel. **A**, **B**, **C**, and **D** Cells of the Caco-2 line were incubated with the indicated concentrations of MC (100 µM), Cur (10 µM), and HC (9 µM) for 48 h, and the expression of nucleoprotein PPARγ (**A**) and the total proteins SP-1 (**B**), SREBP-2 (**C**), NPC1L1 (**D**) were determined by Western blot analysis. Data presented as mean ± SEM. (*n* = 3). ^*^P < 0.05 vs control, ^**^P < 0.01 vs control, ^#^*P* < 0.05 vs MC, ^##^*P* < 0.01 vs MC, ^###^*P* < 0.001 vs MC, ^&^*P* < 0.05 vs MC + Cur, ^&&^*P* < 0.01 vs MC + Cur. MC, cholesterol micelles; Cur, curcumin; HC, HC-030031; PPARγ, peroxisome proliferator-activated receptor gamma; PCNA, proliferating cell nuclear antigen; SP-1, specificity protein-1; GAPDH, glyceraldehyde-3-phosphate dehydrogenase; SREBP-2, sterol regulatory element-binding protein − 2; NPC1L1, Niemann-Pick C1-like 1; TRPA1, transient receptor potential cation channel subfamily A member 1; SEM, standard error of the mean

Furthermore, an *in vitro* model of high cholesterol by treating Caco-2 cells with MC was established. As shown in Fig. 6A, the MC-induced decreased nuclear translocation of PPARγ was promoted at control levels by Cur. However, this activation effect was reversed by HC (Fig. 6A). Moreover, 10 µM of Cur markedly reduced the expression of the SP-1 (Fig. 6B), SREBP-2 (Fig. 6C), and NPC1L1 (Fig. 6D) proteins compared with the group of Caco-2 cells incubated with MC alone, and the inhibitory role was also blocked by HC. This indicated that the TRPA1 channel plays an important role in the process of cholesterol transport.

## Discussion

**Fig. 7:**
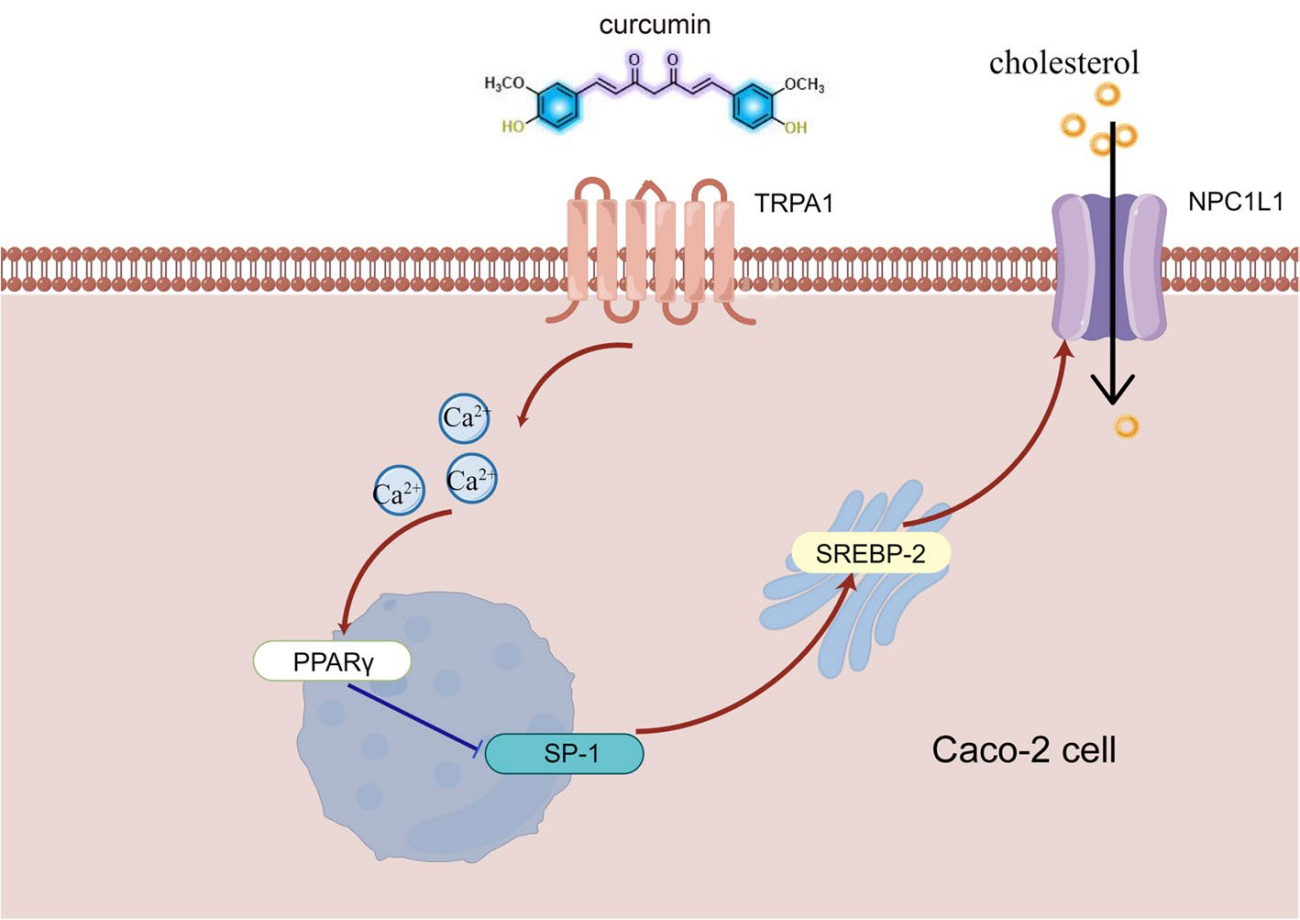
Schematic representation of the reduced cholesterol absorption in Caco-2 cells by Cur. In Caco-2 cells, Cur first stimulates calcium influx by activating the TRPA1 channel, further upregulates PPARγ and downregulates SP-1/SREBP-2/NPC1L1 signaling pathway, and finally inhibits the absorption of cholesterol. TRPA1, transient receptor potential cation channel subfamily A member 1; NPC1L1, Niemann-Pick C1-like 1; PPARγ, peroxisome proliferator-activated receptor gamma; SP-1, specificity protein-1; SREBP-2, sterol regulatory element-binding protein-2; Cur, curcumin

CRC is a major threat to human health, and lipid homeostasis in the gut is closely associated with CRC. Cur could inhibit Caco-2 cells proliferation and reduce cholesterol absorption. However, the specific targets of those effects remain unclear. In this study, a novel function of the TRPA1 channel and its potential mechanism inhibiting cholesterol absorption and cell proliferation in the biology of Caco-2 cells were characterized. This *in vitro* study demonstrated that the TRPA1 channel-mediated the effect of Cur in reducing cholesterol absorption in human Caco-2 cells, and this effect of Cur was determined by regulation of the Ca^2+^/PPARγ/SP-1/SREBP-2/NPC1L1 signaling pathway. Moreover, this receptor also mediated the antiproliferative effect of Cur in a high-lipid environment (Fig. 7).

A variety of colorectal cancer cell lines have shown differential expression of genes related to the proposed pathways [29]. Expression of *NPC1L1*, *TRPA1*, *PPARγ*, *SP-1*, and *SREBP-2* were obtained from Expression Atlas (https://www.ebi.ac.uk/gxa/home) and normalized by fragments per kilobase of transcript per million fragments mapped (FPKM) or transcripts per million (TPM). After searching Expression Atlas, *NPC1L1* was found to be highly expressed in Caco-2 and SW620 cell line, moderately expressed in LoVo cell line, and poorly expressed in HCT116, SW480, and HF-29 cell line [28] (Supplemental Fig. 2A). Further, *TRPA1, PPARγ, SP-1, SREBP-2* were differently expressed in Caco-2, SW620, HCT116, SW480, LoVo, and HF-29 cell line [29] (Supplemental Fig. 2B). Moreover, Cur inhibited proliferation of Caco-2, SW620, HCT116, and LoVo cells to varying degrees, but only regulated intracellular cholesterol content in Caco-2 cells (Supplemental Fig. 3). It is likely that the signal related to the TRPA1 channel is not the only pathway that contributes to the proliferation of these cell lines. Therefore, the dissimilar anti-proliferative mechanisms of Cur need to be explored in the future on SW620, HCT116, and LoVo cell line that have different expressions of the proposed pathway. In addition, *TRPA1* was rarely expressed in SW620 cell line [6] (Supplemental Fig. 2B), which explains the failure of curcumin to inhibit cholesterol absorption in SW620 cell line. To summarize, both NPC1L1 and the TRPA1 channel likely participate in mediating the inhibitory effect of curcumin on cholesterol absorption in Caco-2 cell line.

The anti-tumoral properties of Cur have been attributed to its ability to modulate the pathways and cell cycle involved in proliferation, migration, apoptosis, angiogenesis invasion, and metastasis, or to its role as an antioxidant and anti-inflammatory agent [15]. Similarly, Cur exerts several antitumor properties against CRC acting through a variety of cellular signaling pathways [18]. Apoptosis suppression, cell division inhibition, and cycle activation are all related to antiproliferative properties of Cur against CRC cell lines [11]. In a clinical study, curcuminoids were found to improve the functional scales and global quality of life in CRC patients [39]. Meanwhile, various in vitro and in vivo studies have used Cur in combination with classical chemotherapy, targeted therapies, radiotherapy, and immune checkpoint inhibitors used against CRC [18]. In conclusion, Cur may be used as an adjuvant in CRC treatment.

TRPA1, a member of the transporter transient receptor potential family, is a non-selective and Ca^2+^-permeable cation channel. This channel could transduce noxious chemical stimuli into neuropeptide release and nociceptor electrical excitation, leading to neurogenic inflammation and pain [40]. TRPA1 channels are widely distributed in the intestinal tract [41] and other tissues [42], and may play a key role in various cancers [43–45]. In addition, Cur was shown to activate TRPA1 channels in HEK293 cells [23]. A study discovered that Cur could decrease oral carcinoma CAL-27 cells viability owing to the apoptotic activation associated with Notch-1 and NF-κB downregulation [46]. However, this study failed to explain why Cur could inhibit cell proliferation in a high-lipid environment. In the present study, Cur inhibited the proliferation of Caco-2 cells by activating the TRPA1 channel in a high-lipid environment. The TRPA1 channel emerges as a potential novel therapeutic target for Cur used to treat CRC. However, further studies are needed to investigate the possible signaling pathways involved.

Increased dietary [47] and serum cholesterol [48, 49] are risk factors for CRC, and the latest research suggested that statins, which lower cholesterol through inhibiting cholesterol synthesis, may be related to increased CRC risk [50]. Moreover, a previous study has shown that increased cholesterol levels in CRC tissues can promote the proliferation of IECs and tumor development[51], suggesting that inhibition of cholesterol absorption has potential indirect antitumor effects. JinFeng Zhao *et al.* found that inhibition of the TRPA1 channel in vascular smooth muscle could downregulate the expression of cholesterol efflux transporter ABCA1, thereby decreasing cholesterol efflux [52]. Similarly, based on the current study, the TRPA1 channel mediated the potential indirect antitumor effect of Cur in reducing cholesterol absorption by regulating intracellular cholesterol transport NPC1L1 on human Caco-2 cells.

The present study shows that the effect of Cur in cholesterol absorption was determined by regulating the Ca^2+^/PPARγ/SP-1/SREBP-2/NPC1L1 signaling pathway. Previously, studies have demonstrated that PPARγ, SP-1, SREBP-2, and NPC1L1 were involved in the regulation of CRC cell proliferation, migration, and invasion [53–56]. First, a study has revealed that the natural compound hydroxysafflor yellow A could inhibit the migration, invasion, and proliferation of CRC cells via activation of PPARγ/phosphatase and tensin homolog/Akt signaling [53]. Similarly, PPARγ activation by 10 µM BRL enhanced the anti-proliferative effects; nevertheless, PPARγ inhibition by 5 µM GW 9662 efficiently blocked this effect of Cur on Caco-2 cells in the current study (Supplemental Fig. 4). In conclusion, the inhibitory effect of Cur on Caco-2 cell proliferation was related to the activation of PPARγ. Second, Cur could execute anti-metastatic effects via downregulation of SP-1, CD24, and focal adhesion kinase and by upregulation of E-cadherin expression in CRC cells [54]. Finally, as the high expression of NPC1L1 was related to CRC development, prognosis, and pathological stage [55], and Jianming *et al.* reported that NPC1L1 knockdown in a murine model of colitis-associated CRC resulted in the decreased number of tumors [56]. In addition, NPC1L1 expression was significantly elevated in human CRC tissues from the TCGA database in the current study. Those cholesterol transport-related proteins might play roles in therapeutic targeting on the high-fat induced CRC. More work must be done to resolve this issue. Furthermore, NPC1L1 expression is mainly modulated by SP-1/SREBP-2 [30]. Recently, a study has reported that the activation of PPARγ potentially mediated the Cur-caused suppression of the gene expression of SP-1, leading to the decrease in the SREBP-2 promoter activity and the inhibition of SREBP-2 expression in HSCs [20]. Similarly, this study also discovered the involvement of PPARγ in inhibiting the expression of SP-1 and SREBP-2 in Caco-2 cells. However, the expression of a gene can be regulated at the transcriptional, post-transcriptional, and post-translational level [57]. This study aimed to identify differentially expressed proteins and pave the way for further study to confirm the potential regulating level of the proposed signaling protein expression.

The use of Cur is limited by its poor bioavailability and poor water solubility, poor bioactive absorption, physicochemical instability, rapid metabolization, and low pharmacokinetics [58]. However, these limitations could be conquered by novel approaches, such as nanoencapsulation of Cur [59]. Experiments have demonstrated that nanoforms of curcumin are effective in the treatment of CRC [59]. Since Cur and nano-curcumin formulations have shown beneficial antitumor effects on CRC, large prospective studies are urgently needed to confirm this preclinical evidence.

### Comparisons with other studies and what does the current work add to the existing knowledge

Previous studies have revealed the relation between intestinal cholesterol homeostasis and CRC. Moreover, studies have demonstrated the potential anti-tumor effects of Cur on CRC. The present study showed that the TRPA1 channel is a key site involved in inhibiting cell proliferation and reducing cholesterol absorption in Caco-2 cells, and the Ca^2+^/PPARγ/SP-1/SREBP-2/NPC1L1 signaling pathway is a potential mechanism related to the effects of Cur on the cholesterol absorpting inhibition.

### Study strengths and limitations

The study has several strengths. The present study focused on intestinal lipid metabolism, and explored the molecular mechanisms involved in Cur-induced suppression of cell proliferation and reduction cholesterol absorption in Caco-2 cells. Moreover, this study showed a potential role of Cur in the primary prevention of CRC, and identified the TRPA1 channel as a promising therapeutic target for CRC.

However, some limitations of our study should be considered while interpreting the results. The first limitation is the non-specific bands detected in Western blot raw images, which are possibly due to non-specific binding of the polyclonal antibodies, and likely resulted from non-specific crosslinking or non-specific association of these protein subunits upon denaturation. Another limitation is that drug off-target effects may lead to unintended side effects or unwanted toxicity [60], and HC is at risk of off-target effects as with any small molecule inhibitor, which may affect the final results. Further studies involving siRNA inhibition and overexpression of TRPA1 will provide more definitive evidence.

## Conclusions

In summary, the present study demonstrates that Cur suppresses the proliferation of Caco-2 cells and reduces cholesterol absorption through activate the TRPA1 channel. Furthermore, the potential antitumor effect of Cur in a high-lipid environment was determined by regulating the Ca^2+^/PPARγ/SP-1/SREBP-2/NPC1L1 pathway. Cur is a bioactive dietary polyphenol that exhibits anticancer and lipid-lowering effects in vitro. A drug targeting this channel might be a promising therapeutic option for the treatment of CRC, and Cur could serve as a natural active ingredient to be used in the primary prevention of CRC in clinical practice.

## Supplementary Information

**Additional file 1: Supplemental Fig 1. **Effect of Cur on Caco-2 cell proliferation assessed by inverted microscopy (magnification, 400×). **A **Caco-2 cells were incubated with different concentrations of Cur (0, 5, 10, 20, 50, and 100 μM) for 48 h. **B** Cells of the Caco-2 line were incubated with the indicated concentrations of MC (100 μM), Cur (10 μM), and HC (9 μM) for 48 h. Cur, curcumin; MC, micellar cholesterol; HC, HC-030031. **Supplemental Fig 2. **Expression levels of *NPC1L1*, *TRPA1*, *PPARγ*, *SP-1*, *SREBP-2* in the colorectal cancer cell lines (taken from the Expression Atlas [https://www.ebi.ac.uk/gxa/home]). **A** Expression levels of *NPC1L1* in the colorectal cancer cell lines. **B** Expression levels of *TRPA1*, *PPARγ*, *SP-1*, *SREBP-2* in the Caco-2, SW620, LoVo, HCT116, SW480, and HF-29 cell line. FPKM, fragments per kilobase of transcript per million fragments mapped; NPC1L1, Niemann-Pick C1-like 1; TPM, transcripts per million; TRPA1, transient receptor potential cation channel subfamily A member 1; PPARγ, peroxisome proliferator-activated receptor gamma; SREBP-2, sterol regulatory element-binding protein-2; SP-1, specificity protein-1. **Supplemental Fig 3. **The potential effect of Cur on Caco-2, SW620, HCT116, and LoVo cell lines. **A** and **B** Caco-2, SW620, HCT116, and LoVo cells were incubated with 10 μM Cur for 48 h. Cell viability was assessed using CCK-8 assay (**A**). Effect of Cur on cellular uptake of micellar cholesterol in Caco-2, SW620, HCT116, and LoVo cell line (**B**). Data presented as mean ± SEM. (*n* = 4). **P < 0.01 vs control, ***P < 0.01 vs control (**A**) or MC (**B**). Cur, curcumin; MC, micellar cholesterol; CCK-8, Cell Counting Kit-8; SEM, standard error of the mean. **Supplemental Fig 4. **Effect of the PPARγ signal on Caco-2 cell proliferation with Cur.  Caco-2 cells were pretreated with 10 μM BRL or 5 μM GW9662 and incubated with 10 μM Cur for 48 h. Cell viability was assessed using CCK-8 assay. Data presented as mean ± SEM. (*n* = 5). ^***^P < 0.001 vs control, ^###^*P* < 0.001 vs Cur, ^&&^*P* < 0.01 vs Cur. Cur, curcumin; BRL, rosiglitazone, BRL 49653; GW, GW 9662; PPARγ, peroxisome proliferator-activated receptor gamma; CCK-8, Cell Counting Kit-8; SEM, standard error of the mean.

**Additional file 2.**

## Data Availability

All data related to this study are available upon request.

## Abbreviations

Cur: Curcumin
CRC: Colorectal cancer
TRPA1: Transient receptor potential cation channel subfamily A member 1
PPARγ: Peroxisome proliferator-activated receptor gamma
SP-1: Specificity protein-1
SREBP-2: Sterol regulatory element-binding protein-2
NPC1L1: Niemann-Pick C1-like 1
IECs: Intestinal epithelial cells
HSCs: Hepatic stellate cells
DMEM: Dulbecco's Modified Eagle Medium
CCK-8: Cell Counting Kit-8
EdU: 5-Ethynyl-2′-deoxyuridine
PBS: Phosphate-buffered saline
BCA: Bicinchoninic acid
Cyclin D1: Cyclin dependent kinase 1
TBST: Tris-buffered saline with Tween-20
TCGA: The Cancer Genome Atlas
COAD: Colon adenocarcinoma
COADREAD: Colon adenocarcinoma/rectum adenocarcinoma/esophageal carcinoma
SEM: Standard error of the mean
IC_50_: Inhibitory concentration 50
MC: Micellar cholesterol
HC: HC-030031
AUC: Area under curve
BRL: Rosiglitazone, BRL 49653
FPKM: Fragments per kilobase of transcript per million fragments mapped
TPM: Transcripts per million
