# Supplementary material for: Curcumin suppresses cell proliferation and reduces cholesterol absorption in Caco-2 cells by activating the TRPA1 channel
Source: Lipids Health Dis. 2023 Jan 14;22:6. doi: 10.1186/s12944-022-01750-7 (PMC9840307; doi:10.1186/s12944-022-01750-7)
Supplement: Supplementary file 1 — Additional file 1: Supplemental Fig 1. Effect of Cur on Caco-2 cell proliferation assessed by inverted microscopy (magnification, 400×). A Caco-2 cells were incubated with different concentrations of Cur (0, 5, 10, 20, 50, and 100 μM) for 48 h. B Cells of the Caco-2 line were incubated with the indicated concentrations of MC (100 μM), Cur (10 μM), and HC (9 μM) for 48 h. Cur, curcumin; MC, micellar cholesterol; HC, HC-030031. Supplemental Fig 2. Expression levels of NPC1L1, TRPA1, PPARγ, SP-1, SREBP-2 in the colorectal cancer cell lines (taken from the Expression Atlas [https://www.ebi.ac.uk/gxa/home]). A Expression levels of NPC1L1 in the colorectal cancer cell lines. B Expression levels of TRPA1, PPARγ, SP-1, SREBP-2 in the Caco-2, SW620, LoVo, HCT116, SW480, and HF-29 cell line. FPKM, fragments per kilobase of transcript per million fragments mapped; NPC1L1, Niemann-Pick C1-like 1; TPM, transcripts per million; TRPA1, transient receptor potential cation channel subfamily A member 1; PPARγ, peroxisome proliferator-activated receptor gamma; SREBP-2, sterol regulatory element-binding protein-2; SP-1, specificity protein-1. Supplemental Fig 3. The potential effect of Cur on Caco-2, SW620, HCT116, and LoVo cell lines. A and B Caco-2, SW620, HCT116, and LoVo cells were incubated with 10 μM Cur for 48 h. Cell viability was assessed using CCK-8 assay (A). Effect of Cur on cellular uptake of micellar cholesterol in Caco-2, SW620, HCT116, and LoVo cell line (B). Data presented as mean ± SEM. (n = 4). **P < 0.01 vs control, ***P < 0.01 vs control (A) or MC (B). Cur, curcumin; MC, micellar cholesterol; CCK-8, Cell Counting Kit-8; SEM, standard error of the mean. Supplemental Fig 4. Effect of the PPARγ signal on Caco-2 cell proliferation with Cur. Caco-2 cells were pretreated with 10 μM BRL or 5 μM GW9662 and incubated with 10 μM Cur for 48 h. Cell viability was assessed using CCK-8 assay. Data presented as mean ± SEM. (n = 5). ***P < 0.001 vs control, ###P < 0.001 vs Cur, && [file 12944_2022_1750_MOESM1_ESM.doc]

Supplement Table 1.The raw data about the band gray values of figure 5D.

| Cur (μM) | GAPDH | NPC1L1 | NPC1L1/GAPDH |
| --- | --- | --- | --- |
| 0 | 21054.5 | 10942.72 | 0.519733 |
| 5 | 19193.38 | 9464.255 | 0.4931 |
| 10 | 18563.26 | 10361.38 | 0.558166 |
| 20 | 16650.13 | 7383.962 | 0.443478 |
| 50 | 17298.01 | 6541.841 | 0.378185 |
| 100 | 20605.96 | 7195.766 | 0.349208 |
| 0 | 19611.43 | 13978.21 | 0.712758 |
| 5 | 18323.72 | 12422.01 | 0.67792 |
| 10 | 18401.38 | 11732.55 | 0.637591 |
| 20 | 19348.96 | 12103.606 | 0.625543 |
| 50 | 19195.72 | 8802.912 | 0.458587 |
| 100 | 34247.08 | 13103.55 | 0.382618 |
| 0 | 31732.37 | 18299.37 | 0.576678 |
| 5 | 27116.81 | 14392.44 | 0.530757 |
| 10 | 31504.93 | 15935.1 | 0.505797 |
| 20 | 23853.69 | 11677.49 | 0.489546 |
| 50 | 25757.4 | 11099.2 | 0.430913 |
| 100 | 35474.5 | 14219.35 | 0.400833 |

Cur, curcumin; GAPDH, Glyceraldehyde-3-phosphate dehydrogenase; NPC1L1, Niemann-Pick C1-like 1.

Supplement Table 2. The raw data about the band gray values of figure 5E.

| BRL (μM) | GAPDH | SP-1 | SP-1/GAPDH |
| --- | --- | --- | --- |
| 0 | 19250.38 | 9397.205 | 0.488157 |
| 5 | 19462.38 | 7435.719 | 0.382056 |
| 10 | 17234.96 | 6381.841 | 0.370285 |
| 15 | 17114.84 | 5569.719 | 0.325432 |
| 0 | 17950.43 | 11128.03 | 0.619931 |
| 5 | 18679.13 | 12046.01 | 0.644891 |
| 10 | 18029.55 | 10821.55 | 0.600212 |
| 15 | 18129.55 | 7502.719 | 0.413839 |
| 0 | 20600.79 | 14588.33 | 0.708144 |
| 5 | 19779.96 | 10203.67 | 0.515859 |
| 10 | 19098.13 | 8933.891 | 0.467789 |
| 15 | 19284.79 | 7503.79 | 0.389104 |

BRL, rosiglitazone; GAPDH, Glyceraldehyde-3-phosphate dehydrogenase; SP-1, specificity protein-1.

Supplement Table 3. The raw data about the band gray values of figure 6D.

| Group | GAPDH | NPC1L1 | NPC1L1/GAPDH |
| --- | --- | --- | --- |
| Control | 21458.79 | 6755.841 | 0.314829 |
| MC | 20738.67 | 9936.376 | 0.479123 |
| MC/Cur | 20701.96 | 7912.305 | 0.382201 |
| MC/Cur/HC | 20453.01 | 9483.79 | 0.463687 |
| Control | 20873.08 | 7374.77 | 0.353315 |
| MC | 20050.06 | 8572.255 | 0.427543 |
| MC/Cur | 21241.08 | 8001.426 | 0.376696 |
| MC/Cur/HC | 21122.01 | 10807.62 | 0.511676 |
| Control | 21137.79 | 8592.305 | 0.40649 |
| MC | 21285.43 | 9823.619 | 0.461519 |
| MC/Cur | 21481.26 | 9261.962 | 0.431165 |
| MC/Cur/HC | 22874.33 | 12631.18 | 0.552199 |

GAPDH, Glyceraldehyde-3-phosphate dehydrogenase; NPC1L1, Niemann-Pick C1-like 1; MC, micellar cholesterol; Cur, curcumin; HC, HC-030031.
