# Supplementary material for: Curcumin suppresses cell proliferation and reduces cholesterol absorption in Caco-2 cells by activating the TRPA1 channel
Source: Lipids Health Dis. 2023 Jan 14;22:6. doi: 10.1186/s12944-022-01750-7 (PMC9840307; doi:10.1186/s12944-022-01750-7)

# The whole membranes of all western blots in the manuscript

**Figure 1H**

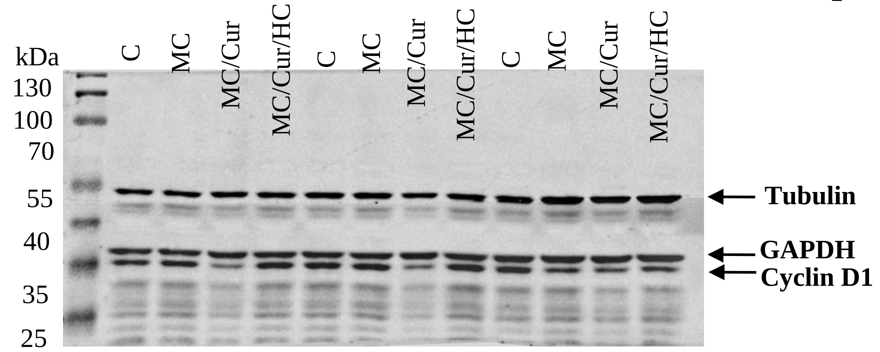


**Figure 4A**

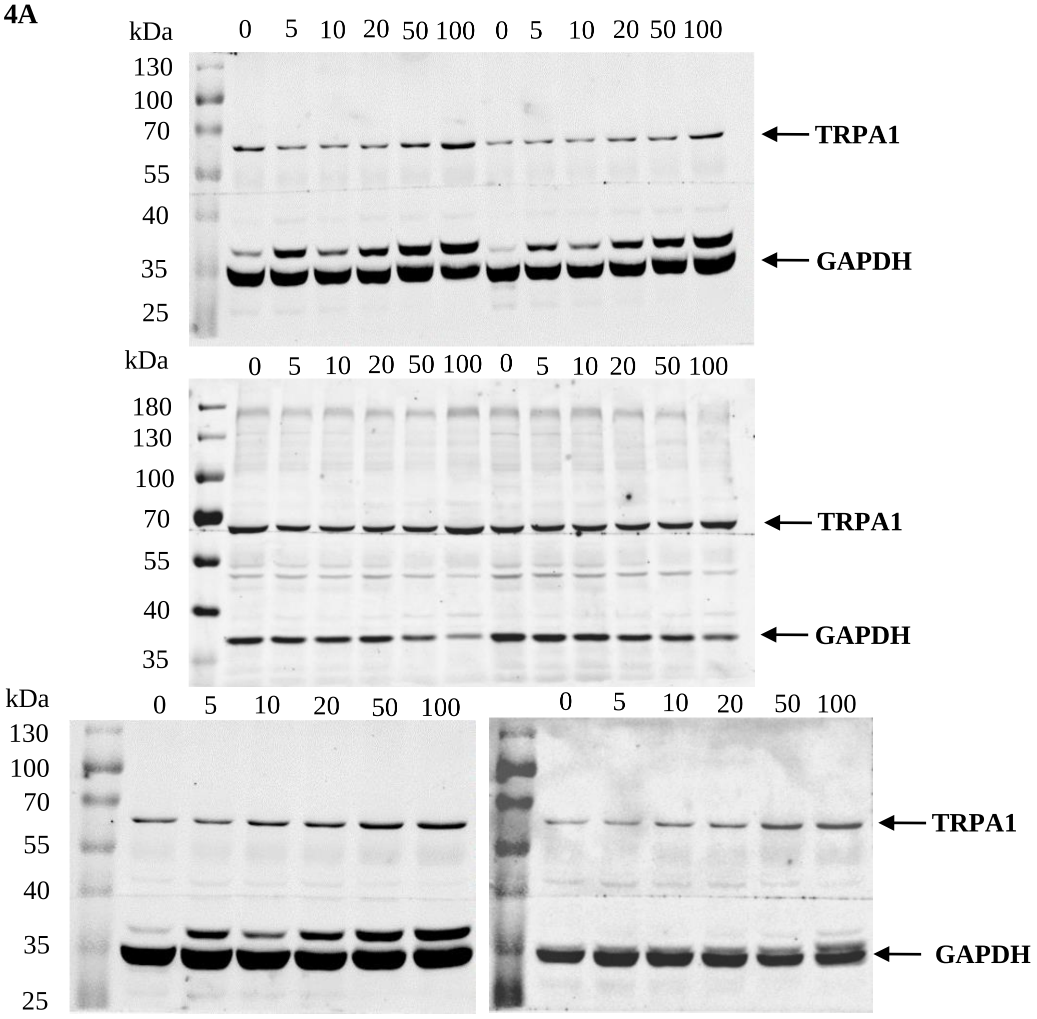


**Figure 4B**

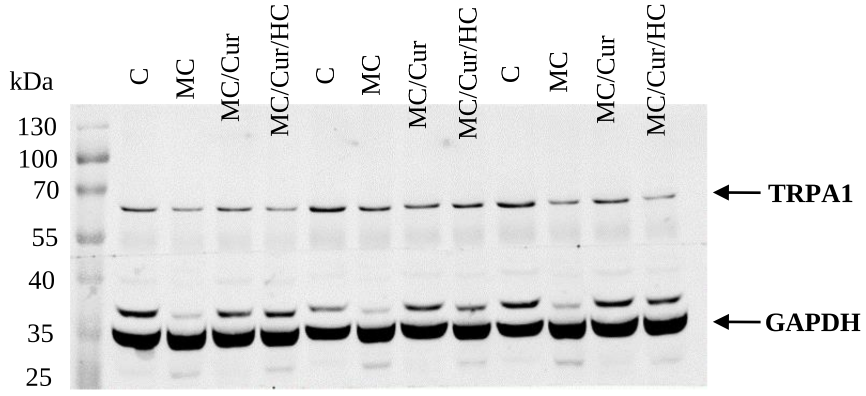

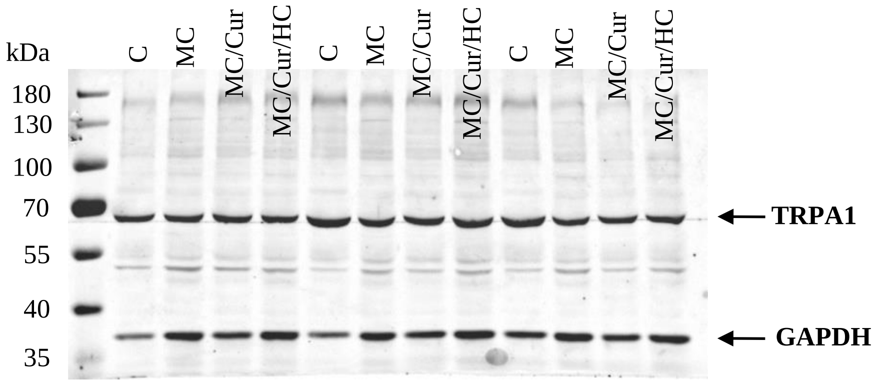


Figure 5B

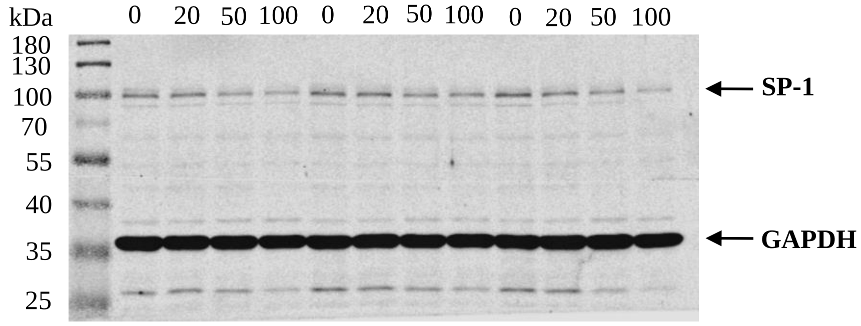


Figure 5C

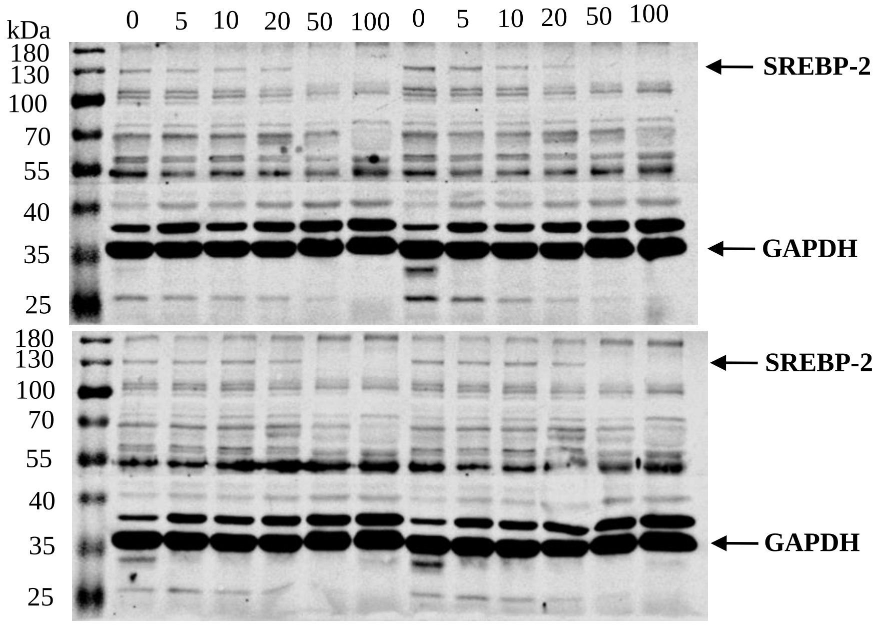


Figure 5D

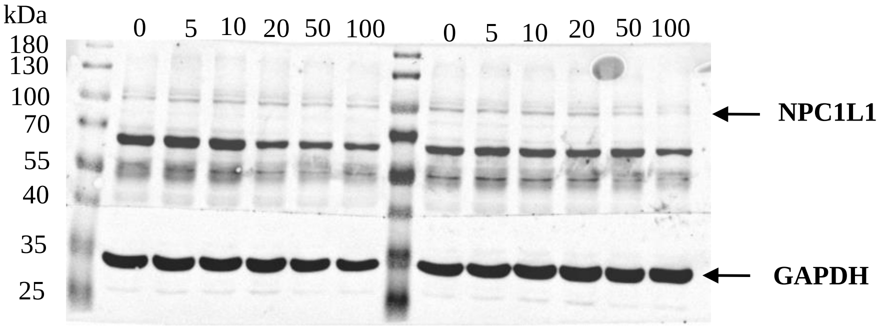

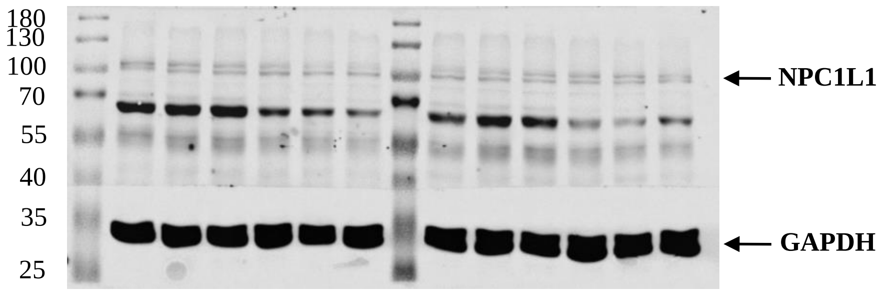


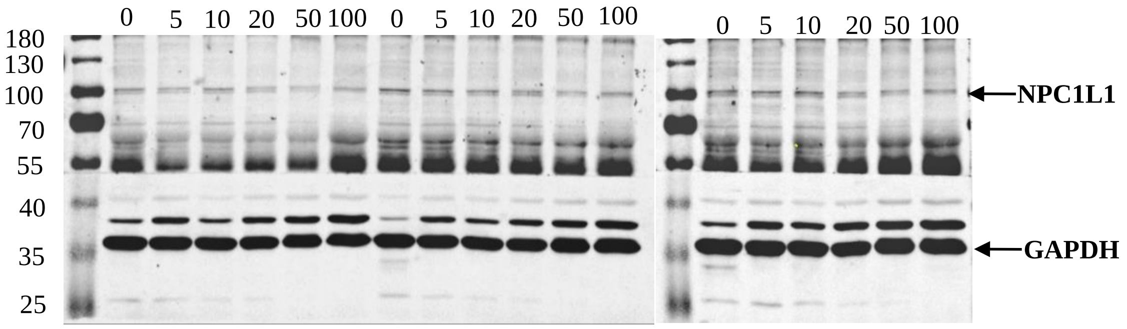


Figure 5E

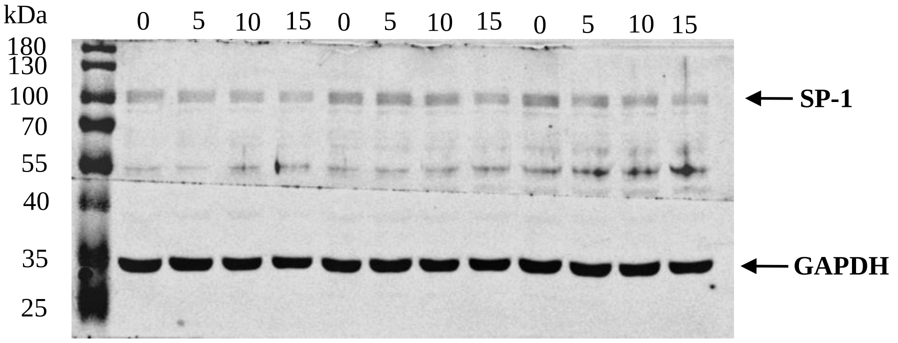


Figure 5F

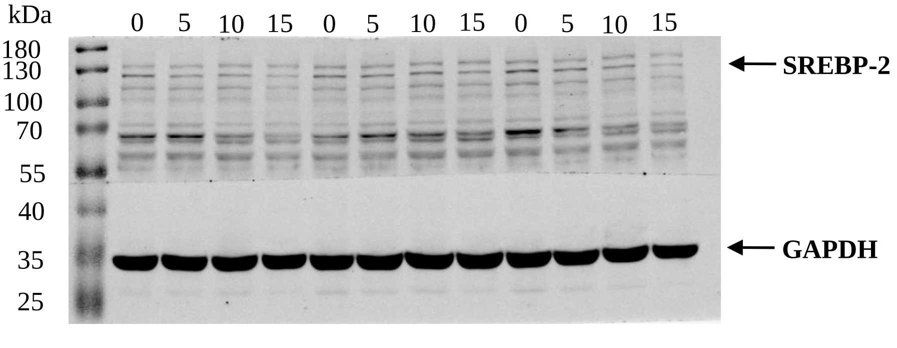

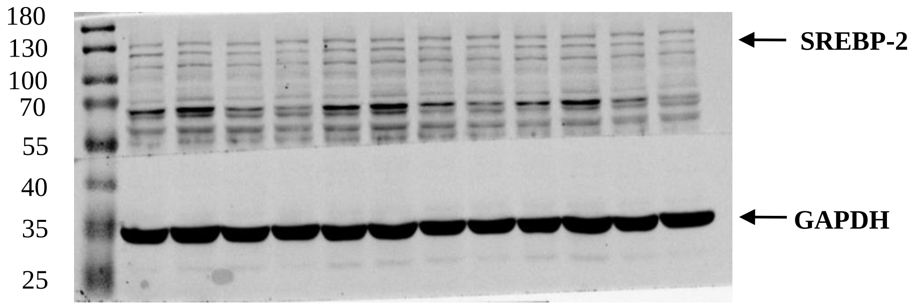


Figure 5G

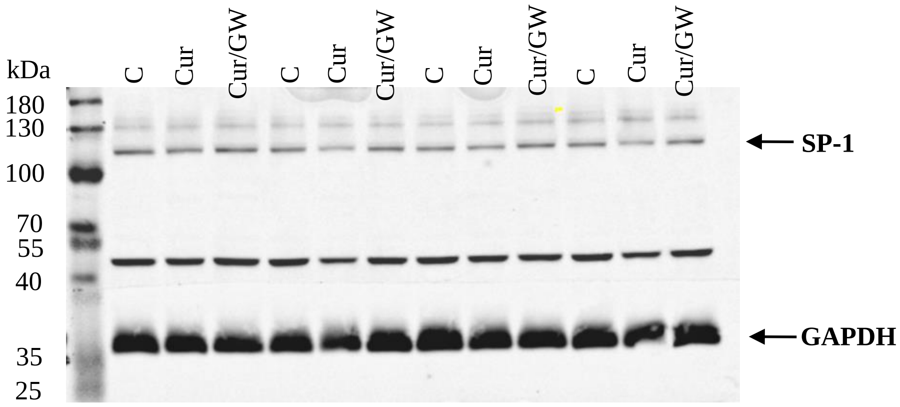


Figure 5H

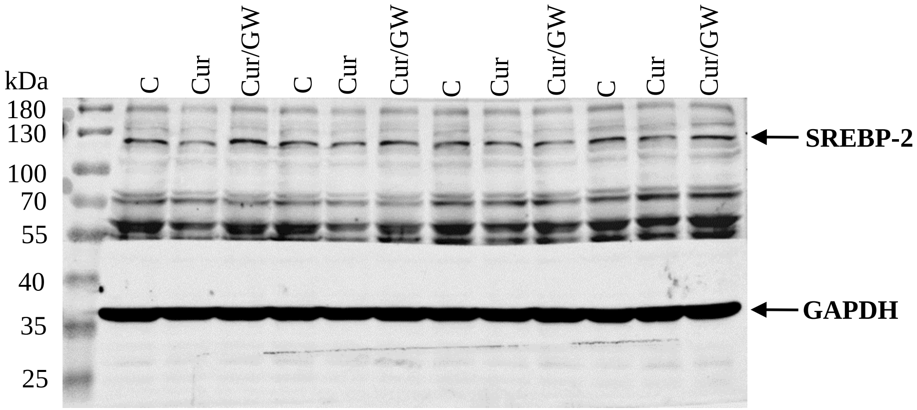


Figure 6A

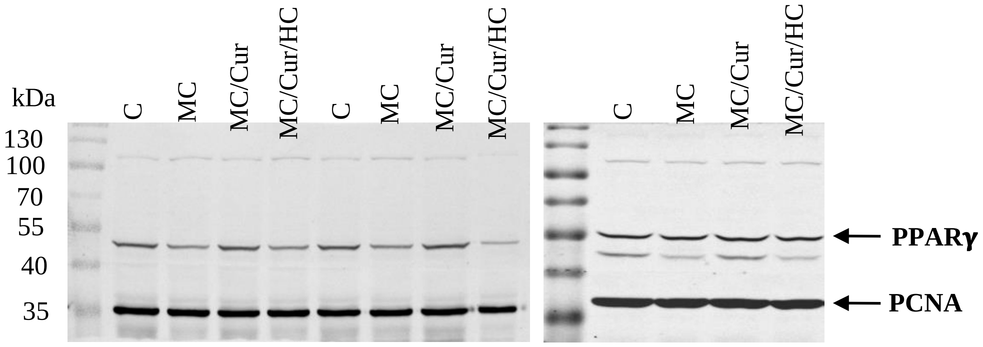

Figure 6B

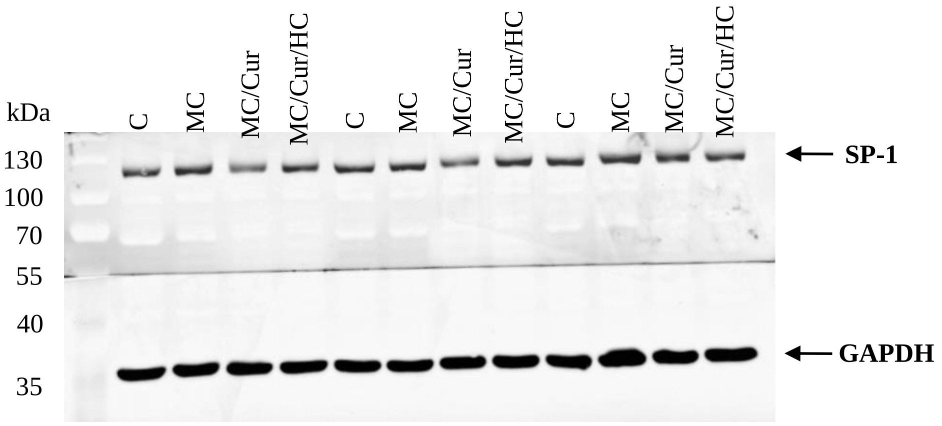


Figure 6C

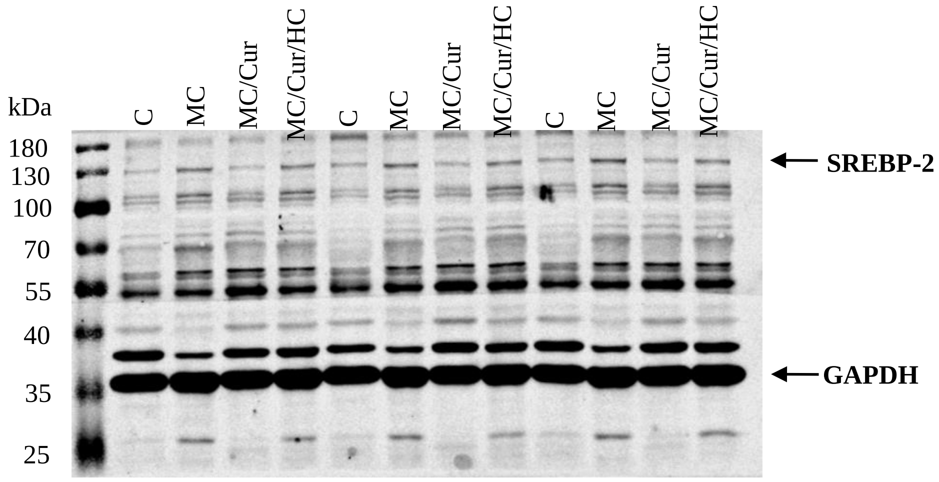


Figure 6D

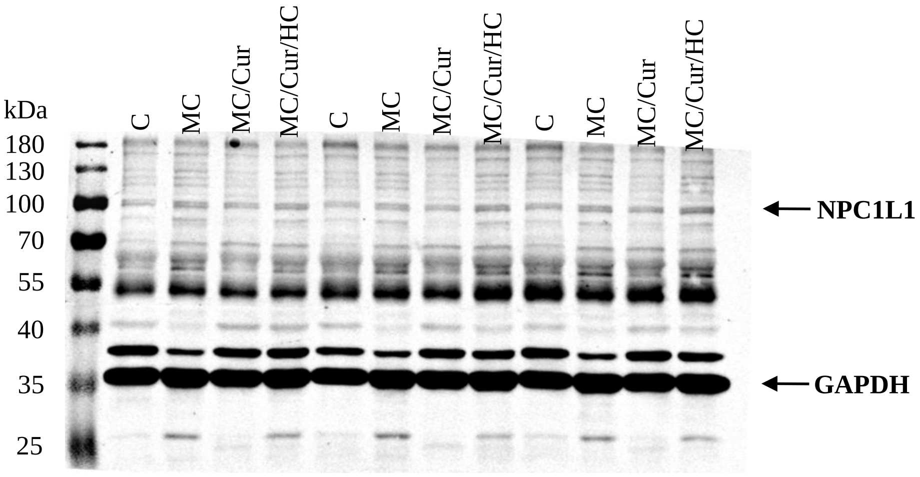

Supplement: Supplementary file 2 — Additional file 2. [file 12944_2022_1750_MOESM2_ESM.pdf]
